# Characterization of pyocyanin with radical scavenging and antibiofilm properties isolated from *Pseudomonas aeruginosa* strain BTRY1

**DOI:** 10.1007/s13205-015-0350-1

**Published:** 2016-01-11

**Authors:** M. Laxmi, Sarita G. Bhat

**Affiliations:** Department of Biotechnology, Cochin University of Science and Technology, Kochi, 682022 Kerala India

**Keywords:** Pyocyanin, Biofilm, FTIR, NMR

## Abstract

A biomolecule namely pyocyanin was isolated from *Pseudomonas aeruginosa* (BTRY1). It was confirmed by UV–Vis spectrum with absorption maxima at 270 nm and was further characterized using Fourier transform infrared (FTIR) and nuclear magnetic resonance (NMR). Spectroscopy analyses showed the presence of relevant bonds in their respective structures. Although pyocyanin had antihemolytic, antioxidant and antibiofilm activities against food pathogens, it showed no observable cytotoxicity, and thus proposed for potential use in the preservation of food.

## Introduction

*Pseudomonas aeruginosa* are Gram-negative, aerobic rod-shaped bacteria, motile by single polar flagellum (Moore et al. 2006); attracting attention due to the different pigments it produces like pyocyanin (blue-green), pyoverdin (yellow, green and fluorescent), pyomelanin (light-brown) and pyorubrin (red-brown) (Meyer 2000). Nearly 90–95 % of *P. aeruginosa* isolates produce pyocyanin, normally referred to as ‘‘blue pus’’ (from pyocyaneus) (Ran et al. 2003). The present study focuses on the extraction, characterization and study of the bioactive properties of the bioactive compound, pyocyanin, isolated from the strain *P. aeruginosa* BTRY1.

## Materials and methods

### Extraction, quantification and characterization of pyocyanin from the strain *Pseudomonas aeruginosa* (BTRY1)

*Pseudomonas aeruginosa* (BTRY1) previously isolated and characterized (Laxmi and Sarita 2014) was inoculated in nutrient broth (HiMedia, Mumbai, India), incubated at 37 °C for 18–24 h and observed for color change, followed by extraction of pyocyanin as per Hayfa et al. (2012). The concentration of pyocyanin was determined (Aziz et al. 2012). Absorption maximum of pyocyanin was measured using UV–visible spectrophotometer (Schimadzu, Japan). Further characterization of pyocyanin was done by Proton Nuclear magnetic resonance spectroscopy (Proton NMR) (Bruker Avance III, 400 MHz) and Fourier Transform infrared spectroscopy (FTIR) (Thermo Nicolet, Avatar 370) (Sudhakar et al. 2013).

### Free radical scavenging activity of pyocyanin

Free radical scavenging activity of pyocyanin was estimated by DPPH radical scavenging assay (Liyana and Shahidi 2005). The results were statistically analyzed after three independent repeats.

### Assay of hemolytic activity and determination of in vitro cytotoxic effect of pyocyanin on cultured L929 cell lines

The hemolytic activity of pyocyanin was evaluated by measuring the release of hemoglobin from fresh human erythrocytes as per Park et al. (2004). L929 fibroblast cell lines (NCCS, Pune, India) was maintained in Dulbecco’s modified Eagles medium (HiMedia) supplemented with 10 % FBS (Invitrogen) and grown to confluence at 37 °C in 5 % CO_2_ in humidified atmosphere of CO_2_ incubator (NBS, Eppendorf, Germany). 3-(4,5-Dimethylthiazol-2-yl)-2,5-Diphenyltetrazolium Bromide (MTT) Assay (Arung et al. 2009) was used to measure cell viability after treatment with pyocyanin at different concentrations to show its cytotoxic effect.

### Antibiofilm activity and determination of biofilm inhibitory concentration (BIC)

The extracted compound was tested for their ability to control biofilm production by strongest biofilm producers in the food industry and with high multiple antibiotic resistance profile (MAR indices) (unpublshd data), taken from the laboratory collection of Department of Biotechnology, CUSAT namely *Vibrio diabolicus* (*TVMS3*) and *Salmonella Enteritidis* (*S49*) (unpublished data) using the standard microtiter plate assay as a semi-quantitative method (Rode et al. 2007). All tests were repeated thrice independently and statistically analyzed (Christensen et al. 1988; Stepanovic et al. 2000). MIC is defined as the lowest concentration of the compound that yields no visible growth. In the case of biofilm formation, it can be defined as the minimum concentration which inhibits biofilm formation that is termed to be biofilm inhibitory concentration (BIC).

## Results

### Extraction, quantification and characterization of pyocyanin from *P. aeruginosa* strain (BTRY1)

**Fig. 1:**
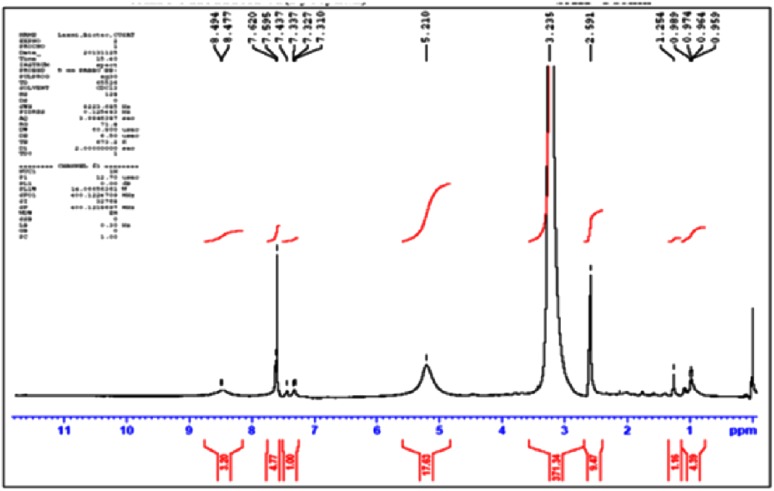
NMR spectrum of pyocyanin isolated from *Pseudomonas aeruginosa* strain BTRY1

**Fig. 2:**
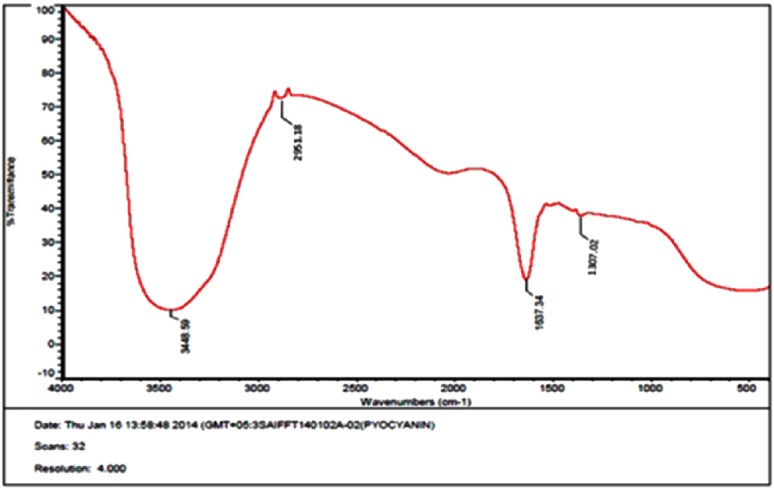
FTIR spectrum of pyocyanin isolated from *Pseudomonas aeruginosa* strain BTRY1

Pyocyanin production after 18 h of incubation was indicated by change in color to bluish green on addition of chloroform, and it was soluble in chloroform. The concentration of pyocyanin was calculated to be 1.245 µg/mL **±** 0.001414. The absorption maximum of the extracted pyocyanin at 270 nm was comparable to that of standard pyocyanin (Sudhakar et al. 2013). The isolated pyocyanin was subjected to ^1^H NMR analysis using cadmium chloride(CdCl_3_) as solvent. In ^1^H NMR (Fig. 1), the peak at δ 2.7–3.4 ppm indicated the presence of methyl group linked to aromatic nitrogen atom. The peak at δ 7.5–7.7 ppm represented the condensed aromatic nitrogen ring. Further characterization of pyocyanin was by analyzing their IR spectrum. The pyocyanin spectrum in Fig. 2 indicated the presence of phenazine as specified by side chains of the molecule. The peak at 3448.59 cm^−1^ shows the presence of O–H bond. The peak at 2951.18 cm^−1^ relates to the C–H– aromatic bond. The peak shown at 1637.34 cm^−1^ represents C=N bond and the peak at 130.7.02 cm^−1^ corresponds to C–O bond. This is comparable to the reports of standard FTIR spectra.

### Free radical scavenging activity of pyocyanin

**Fig. 3:**
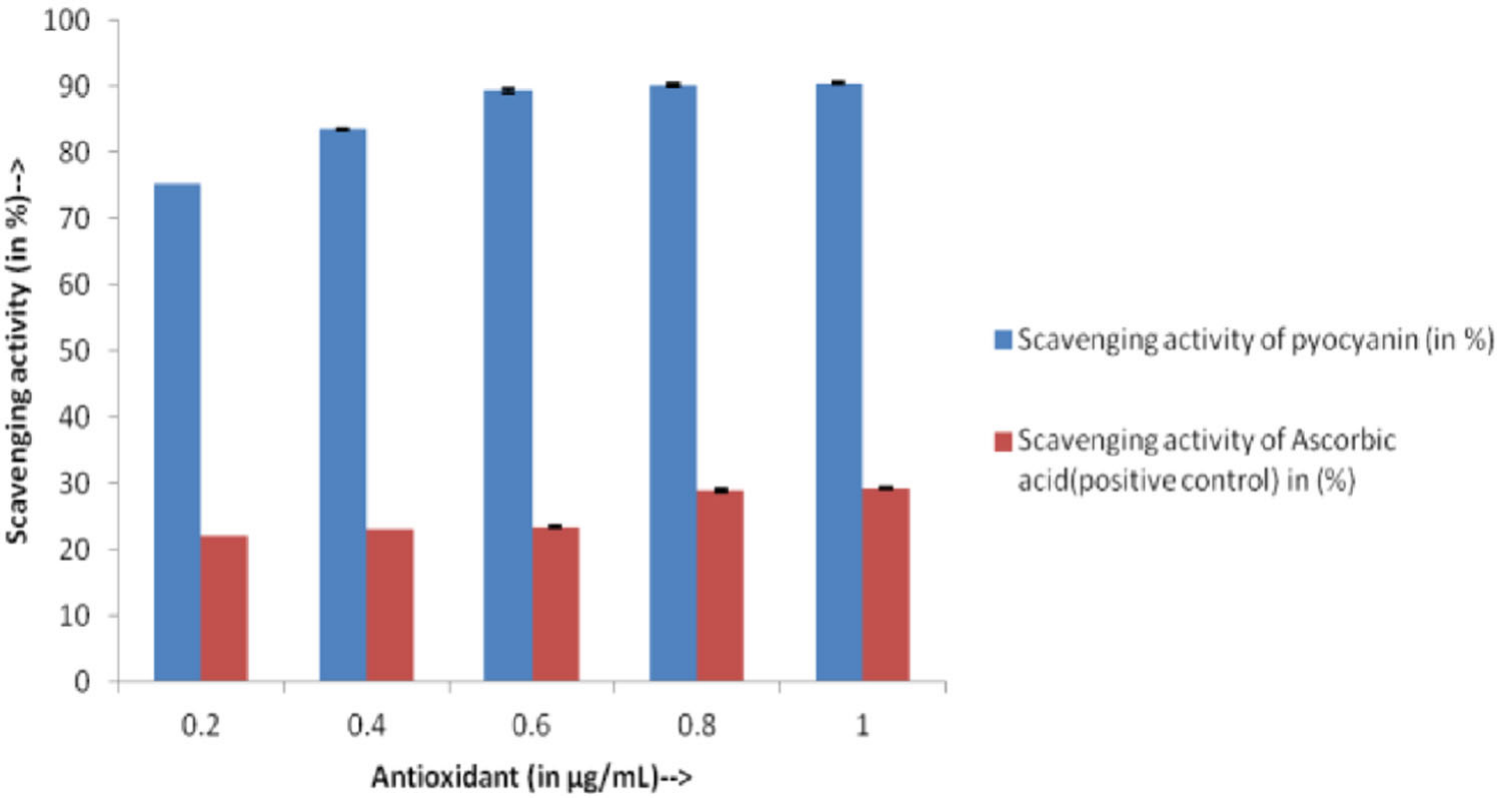
Scavenging activity of pyocyanin isolated from *Pseudomonas aeruginosa* strain BTRY1

Free radical scavenging activity of pyocyanin was much higher than that of ascorbic acid (Fig. 3). The results are significant as higher radical scavenging activities were obtained for pyocyanin (0.2 µg/mL) even at concentration very much lower than that of the ascorbic acid.

### Assay of hemolytic activity and determination of in vitro cytotoxic effect of pyocyanin on cultured L929 cell lines

**Fig. 4:**
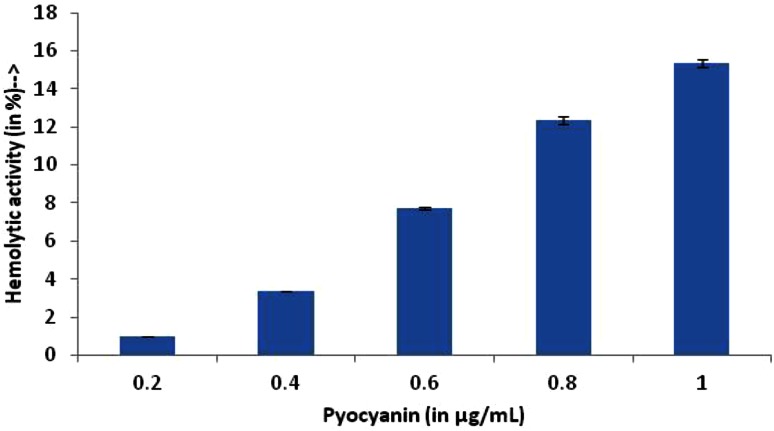
Hemolytic activity of pyocyanin isolated from *Pseudomonas aeruginosa* strain BTRY1

**Fig. 5:**
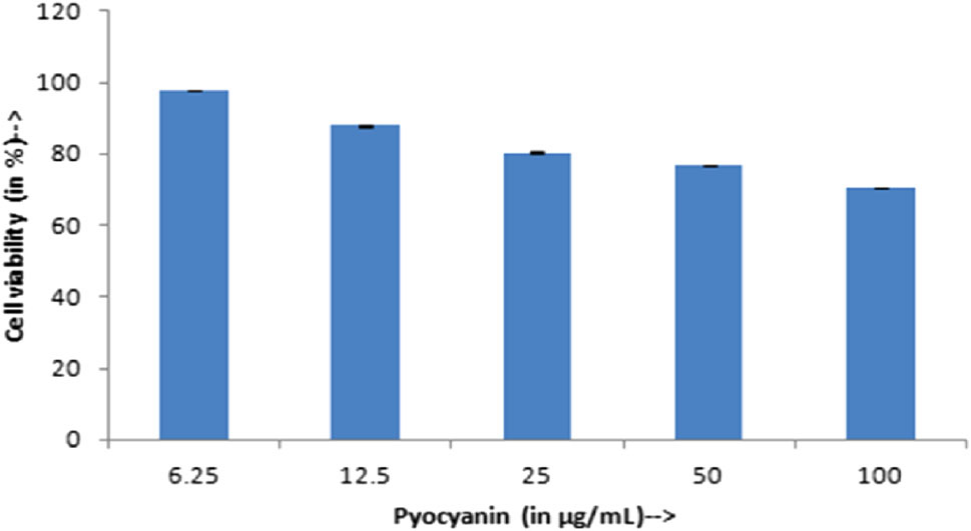
Cyotoxicity of pyocyanin isolated from *Pseudomonas aeruginosa* strain BTRY1

**Fig. 6:**
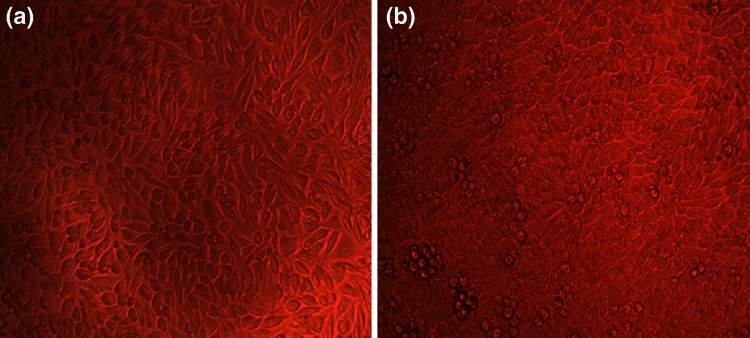
Phase contrast micrographs for cytotoxicity of **a** control and **b** pyocyanin-treated L929 cell lines

Cytotoxicity of pyocyanin was measured by lysis of human erythrocytes. Figure 4 depicts that pyocyanin had reduced hemolytic activity even at higher concentration. At 1 µg/mL for pyocyanin, the hemolytic activity was <16 %, but there was no hemolytic activity at its MIC concentrations. All the results were analyzed with respect to the positive control Triton 100 X, which was considered to be 100 % hemolytic and thus the compound is compared. MTT assay for analyzing the cytotoxic effect of compound on cell lines showed that the compound was not cytotoxic at the tested concentrations. It was observed that the cells showed almost 90 % viability after pyocyanin treatment at 6.25 µg/mL. Figure 5 shows the cell viability of around 80 % even at high concentrations indicating its safety of use in food consumption for humans. Figure 6 shows the phase contrast micrographs showing the viability of cultured L929 cells before and after treatment of pyocyanin.

### Antibiofilm activity and determination of biofilm inhibitory concentration

**Fig. 7:**
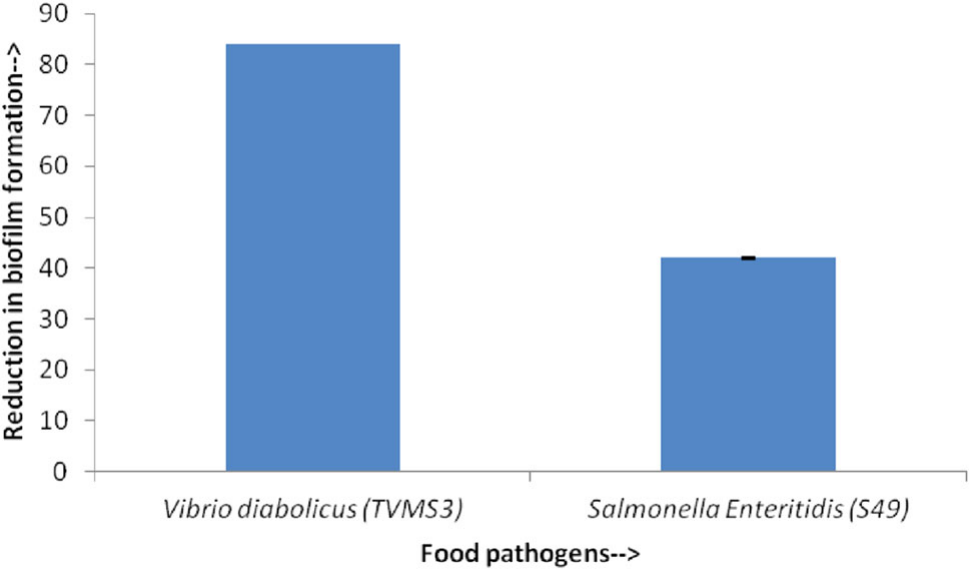
Reduction in biofilm formation (in %) by pyocyanin isolated from *Pseudomonas aeruginosa* strain BTRY1

The antibiofilm activity was tested with pyocyanin at 1.245 µg/mL with serial dilutions thereafter, and the biofilm inhibitory concentration (BIC) for pyocyanin was calculated to be 2 × 10^−2^ ng/µL. The compound caused reduction of biofilm formation by *Vibrio diabolicus* (*TVMS3*) and *Salmonella Enteritidis* (*S49*). Figure 7 shows >80 % reduction of biofilm formation by the test strains.

## Discussion

Pyocyanin, a bio-active compound produced by *Pseudomonas aeruginosa*, was extracted from Pseudomonas broth by chloroform and the blue-green compound was separated. Addition of 0.2 N HCl to obtain a pinkish red colored confirmed the presence of pyocyanin pigment (Raoof and Latif 2010). The separated red color compound on UV-spectrophotometric analysis showed maximum absorption at 270 nm, which is in accordance with the previously published reports (Kerr et al. 1999). It also has the capacity to arrest the electron transport chain of the different microorganisms and to exhibit antimicrobial and antibiofilm activity (Kerr 1994). The FTIR and Proton NMR spectra of the BTRY1 pigment were characteristic of pyocyanin. The compound showed very high free radical scavenging activity at very minute concentrations, which is a positive indication for the safe use of compound (Liyana and Shahidi 2005). In addition to this, the compound showed no cytotoxic effects on human red blood cells (Park et al. 2004) and in cultured L929 cells (Arung et al. 2009). Besides all, the antibiofilm activity of the compound against multiple antibiotic-resistant food pathogens augments their potency for application in food industry. This can be used to control several other potent food pathogens if applied in the food.
